# Small RNA signatures of acute ischemic stroke in L1CAM positive extracellular vesicles

**DOI:** 10.1038/s41598-024-63633-4

**Published:** 2024-06-12

**Authors:** Bharti Manwani, Nivetha Brathaban, Abiya Baqai, Yashee Munshi, Hilda W. Ahnstedt, Mengqi Zhang, Kajsa Arkelius, Ted Llera, Edilberto Amorim, Fanny M. Elahi, Neel S. Singhal

**Affiliations:** 1 Department of Neurology, University of Texas Health Science Center, Houston, TX 77030 USA; 2 Department of Neurology, University of California-San Francisco, San Francisco, CA 94158 USA; 3 Department of Neurology, Icahn School of Medicine at Mount Sinai, New York, NY USA; 4 Department of Neuroscience, Icahn School of Medicine at Mount Sinai, New York, NY USA; 5 James J. Peters Department of Veterans Affairs Medical Center, Bronx, NY USA; 6 Neurology Service, San Francisco Veterans Affairs Medical Center, San Francisco, CA 94150 USA

**Keywords:** Stroke, Biomarker, MicroRNA, Exosome, Extracellular vesicle, Non-coding RNA, Neuroscience, Neurological disorders

## Abstract

L1CAM-positive extracellular vesicles (L1EV) are an emerging biomarker that may better reflect ongoing neuronal damage than other blood-based biomarkers. The physiological roles and regulation of L1EVs and their small RNA cargoes following stroke is unknown. We sought to characterize L1EV small RNAs following stroke and assess L1EV RNA signatures for diagnosing stroke using weighted gene co-expression network analysis and random forest (RF) machine learning algorithms. Interestingly, small RNA sequencing of plasma L1EVs from patients with stroke and control patients (n = 28) identified micro(mi)RNAs known to be enriched in the brain. Weighted gene co-expression network analysis (WGCNA) revealed small RNA transcript modules correlated to diagnosis, initial NIH stroke scale, and age. L1EV RNA signatures associated with the diagnosis of AIS were derived from WGCNA and RF classification. These small RNA signatures demonstrated a high degree of accuracy in the diagnosis of AIS with an area under the curve (AUC) of the signatures ranging from 0.833 to 0.932. Further work is necessary to understand the role of small RNA L1EV cargoes in the response to brain injury, however, this study supports the utility of L1EV small RNA signatures as a biomarker of stroke.

## Introduction

Extracellular vesicles (EVs) positive for L1CAM (L1EVs) have emerged as possible biomarkers of neuronal health, traumatic brain injury, and progression of neurodegenerative disease^1–4^. EVs are released from cells upon fusion of endosomal multivesicular bodies with the plasma membrane^5^. The physiological functions of EVs are incompletely understood, but they have been shown to influence the behavior of distant cell types. Additionally, EVs may serve as biomarkers of physiologic states of their parent cells. Cell-specific EVs can be identified by cell surface proteins, and L1CAM has emerged as the primary marker for presumed neuronally-derived EVs^6^. As L1EVs are continuously turned over in response to physiologic and pathologic stimuli and small RNAs such as micro(mi)RNAs are a major cargo of EVs, small RNA transcriptomic signatures from L1EVs may serve as clinically useful biomarkers of brain injury^7^.

Although no studies have examined miRNA and other noncoding (nc)RNAs with next-generation sequencing techniques in L1EVs after acute ischemic stroke (AIS), some studies have examined miRNA cargos of total circulating exosomes, a subpopulation of EVs, in AIS patients. These studies identified several different miRNA candidates up- and down-regulated in AIS. For example, upregulation of serum exosomal miR-9, miR-124, miR-134, miR-146b, and miR-223 have been correlated with AIS; while decreased expression of serum exosome miR-152-3p has also been observed in another study^8–13^. A more extensive study of 173 patients also found significant increases in miRNA-17-5p, -20b-5p, and -93-5p and miRNA-27b-3p at a 48 h time point following AIS compared to non-stroke patients^14^. Further reinforcing the potential importance of L1EVs as biomarkers of brain injury, in patients suffering from traumatic brain injury (TBI) and cognitive impairment, Goetzl and colleagues found that L1EV cargos such as phosphorylated tau and interleukin-6 were specifically associated with cognitive impairment in TBI^2^.

Micro RNAs (miRNAs) are a class of endogenous, small (~ 17–25 nucleotide), non-coding (nc)RNA that impact cellular function by suppressing or activating downstream mRNA targets, which in turn regulates protein expression. Emerging data is also beginning to shed more light on the pathophysiological roles of miRNAs and other small ncRNAs such as small nucleolar (sno)RNA, transfer (t)RNA, long noncoding (lnc)RNA, circular RNA, yRNA, piwi-interacting RNA, and vaultRNA^15^. To date the most extensive investigations have focused on miRNAs^16^. However, other ncRNAs have been linked to stroke incidence and prevalence in a sub-cohort of the Framingham Heart Study^17^.

Despite numerous studies on blood-based biomarkers of AIS, none are used in routine clinical practice. When coupled with a point-of-care test, blood-based biomarkers of AIS could enable more efficient triage and precise treatment of AIS. Focusing on L1EV small RNAs as biomarkers are promising since they may rapidly cross the blood–brain barrier and thus reflect neuronal damage. In addition, EV-associated RNAs may not be as sensitive to degradation as circulating RNAs^18^. And finally, point-of-care microfluidic devices are being developed to quickly capture specific EV populations^19^.

We performed next-generation small RNA sequencing on L1EVs from plasma of AIS patients and control patients with cardiovascular risk factors. In addition, we applied robust statistical and machine learning algorithms to analyze and provide further insights into the high-dimensional ncRNA expression data, which has not been performed for stroke L1EVs before. We report that L1EV miRNA and ncRNA signatures can accurately distinguish AIS from a non-stroke patient. Our findings provide proof of concept for developing a specific L1EV small RNA signature for AIS. Further understanding L1EV RNA expression patterns acutely following a AIS may also provide insights in the pathophysiological role of neuronally derived EVs following brain injury.

## Results

We performed small RNA sequencing of L1EVs isolated from plasma samples of patients with AIS or patients with cardiovascular risk factors only (Table 1). Consistent EV size and concentrations indicative of high quality EV sample preparation was confirmed across samples with nanoparticle tracking analysis (Supplementary Fig. 1A–C). Small RNA sequencing from L1EVs identified the presence of several ncRNA species including miRNAs (7%), fragments of lncRNA (76%), tRNA (1%), and smaller percentages of vaultRNA, small nuclear (sn)RNA, and snoRNA (< 0.6% of total reads; Supplementary Fig. 1D,E). L1CAM is also expressed in non-neuronal cells^20,21^, however, supporting a neuronal origin of L1EVs, all of the most abundant miRNAs in our sequencing data have previously been described to be expressed in the brain (Supplementary Table 2^22^). Additionally, tissue enrichment analysis demonstrated high degree of enrichment of the 65 most abundant miRNAs in central nervous system tissues^23^ (Supplementary Fig. 1F).

**Table 1:** Patient demographics and outcomes.

	Control (*n* = 12)	Acute ischemic stroke (*n* = 16)
Age, mean (S.D.)	76.2 (6.19)	72.4 (8.24)
Sex (% female)	58.3%	37.5%
Race, (%)		
Black	16.7%	18.8%
Other or multiple races	25%	31.25%
White	58.3%	50%
Hispanic or Latino (%)	25%	25%
Stroke risk factors		
Hypertension	83.3%	87.5%
Atrial fibrillation	16.7%	18.8%
Type 2 diabetes mellitus	33.3%	56.3%
Smoking	25%	18.8%
Hyperlipidemia	58.3%	62.5%
Prior antiplatelet/anticoagulation	75.0%	62.5%
Antiplatelet	75.0%	56.3%
Anticoagulation	0%	6.25%
Stroke type		
Large-artery atherosclerosis	N/A	25%
Cardioembolism	N/A	18.8%
Small-vessel occlusion	N/A	12.5%
Undetermined etiology	N/A	43.8%
Admission NIHSS, median (min, max)	N/A	3 (0–26)
Discharge NIHSS, median (min, max)	N/A	1 (0–9)
Thrombectomy performed (% AIS)	N/A	12.5%
tPA administered (% AIS)	N/A	31.3%
Admission mRS, median (min, max)	0 (0, 0)	0 (0, 4)
Discharge mRS. median (min, max)	0 (0, 0)	3 (0, 5)
Discharged home (%)	100%	75%

**Figure 1:**
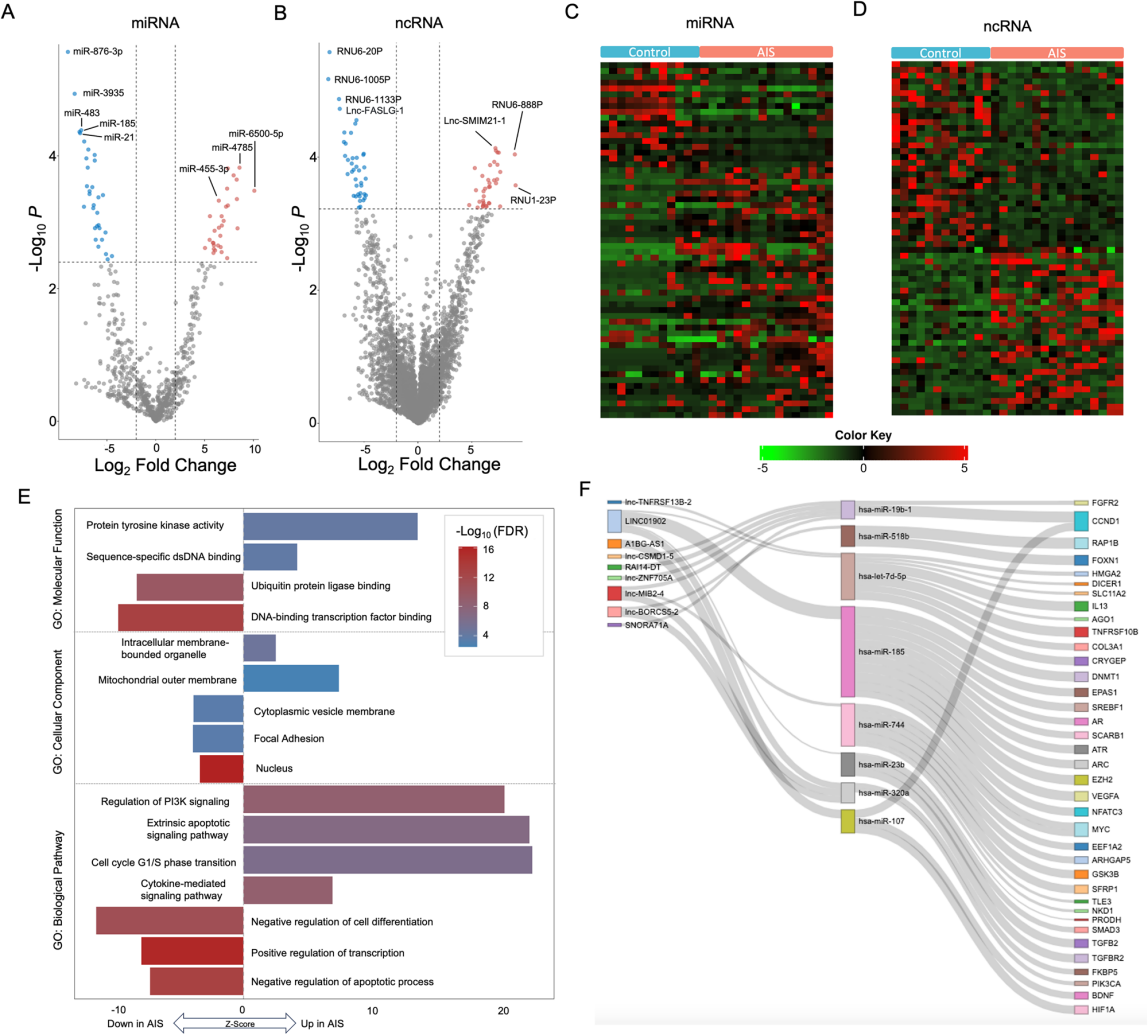
Differential small RNA expression analysis of L1EVs from plasma of acute ischemic stroke (AIS) patients. Volcano plot demonstrating (**A**) miRNAs and other (**B**) ncRNA (i.e. lncRNA, snRNA, snoRNA) significantly regulated following AIS. (**C**,**D**) Heat maps with individual patient relative expression levels of the top differentially expressed up-regulated miRNAs and other ncRNAs after AIS (fold-change [FC] > 2, adjusted P value < 0.05; AIS = 16, non-stroke control = 12). (**E**) Pathway analysis using gene ontology (GO) terms of target genes for the differentially regulated miRNAs reveals significant regulation of miRNAs targeting transcription factors, intracellular signaling, cell death pathways, and inflammatory signaling. (**F**) Sankey plot of the subset of differentially regulated lncRNAs (left column) and miRNAs (middle column) that interact with each other and their potential gene targets (right column). Heatmap generated with iDEP^62^.

We hypothesized that miRNA and other ncRNA profiles from L1EVs reflect states of AIS injury. Supporting this hypothesis, raw L1EV miRNA and other small ncRNA expression counts were produced and normalized, and principal component analysis revealed segregation in sources of variance by AIS diagnosis and sex (Supplementary Fig. 2). Next, we performed differential expression analysis of miRNA and other small ncRNA by diagnosis, revealing 62 miRNA and 76 small ncRNA transcripts that were significantly altered (greater than twofold-change; adjusted P-value < 0.05) in AIS patients (Fig. 1A–D, Supplementary Tables 3, 4). Interestingly, even though snRNAs and snoRNAs made up < 1% of reads they comprised 13% and 6.5% of the differentially expressed ncRNAs. Gene ontology biological pathway analysis of differentially expressed miRNAs revealed enrichment of up-regulated miRNAs involved in regulation of PI3K signaling, apoptotic signaling, and cytokine-mediated signaling (Fig. 1E). Notably, differentially expressed miRNAs were also found to be the target of differentially expressed other ncRNAs and their predicted gene targets included genes with well-known roles in cell death, adaptation to metabolic stressors, and angiogenesis including hypoxia inducible factor (HIF)-1a, FK506 binding protein (FKBP)-5, brain-derived neurotrophic factor (BDNF), and vascular endothelial growth factor (VEGF)-a (Fig. 1F).

**Figure 2:**
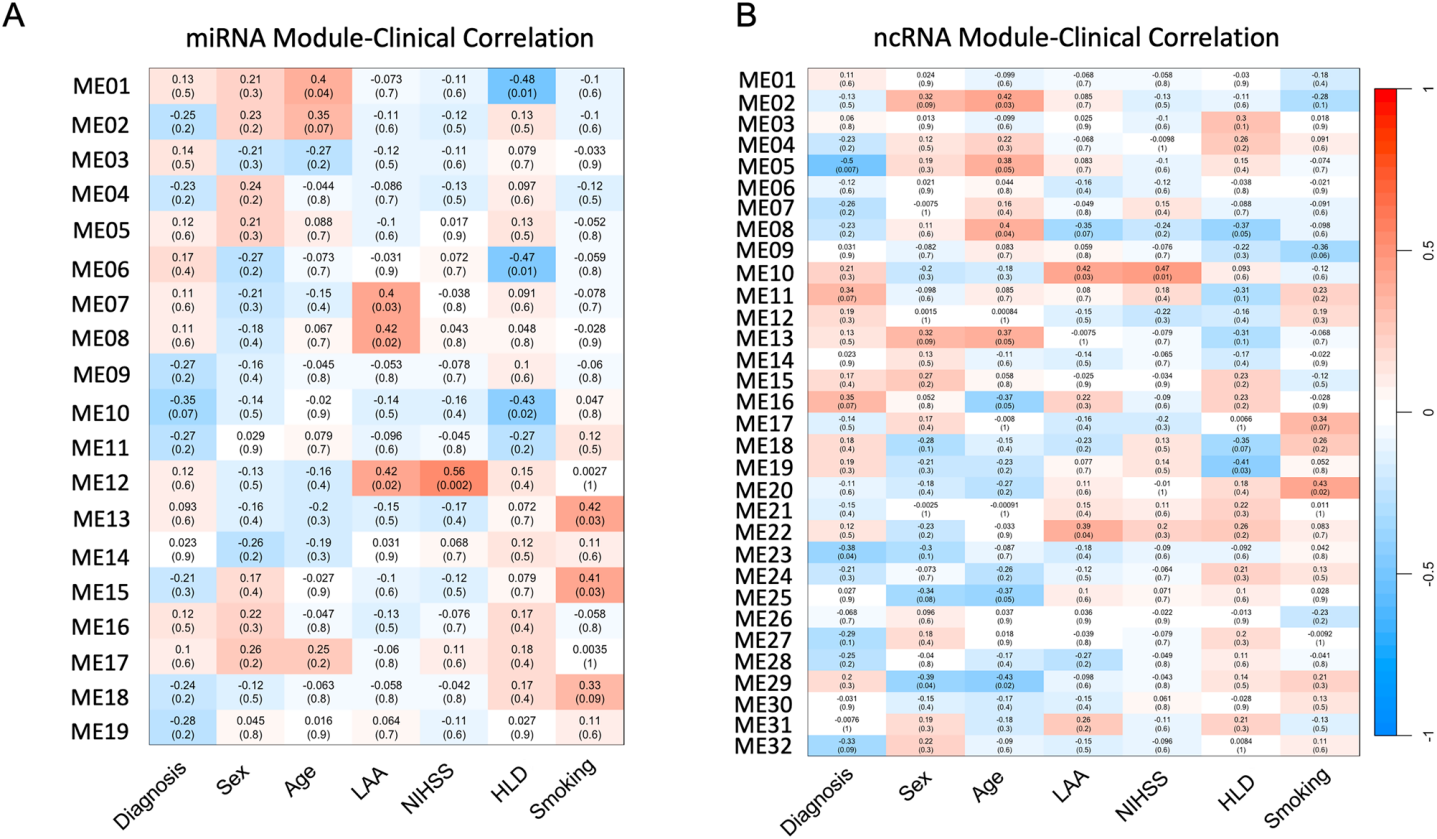
L1EV small RNA co-expression modules correlate with selected clinical variables. (**A**) miRNA and (**B**) other ncRNA with each row corresponding to a module-eigengene (ME) and each column to a clinical variable. Each cell contains the corresponding correlation and p-value and color-coded by correlation. LAA: large artery atherosclerosis stroke subtype; NIHSS: NIH stroke scale; HLD: hyperlipidemia.

**Figure 3:**
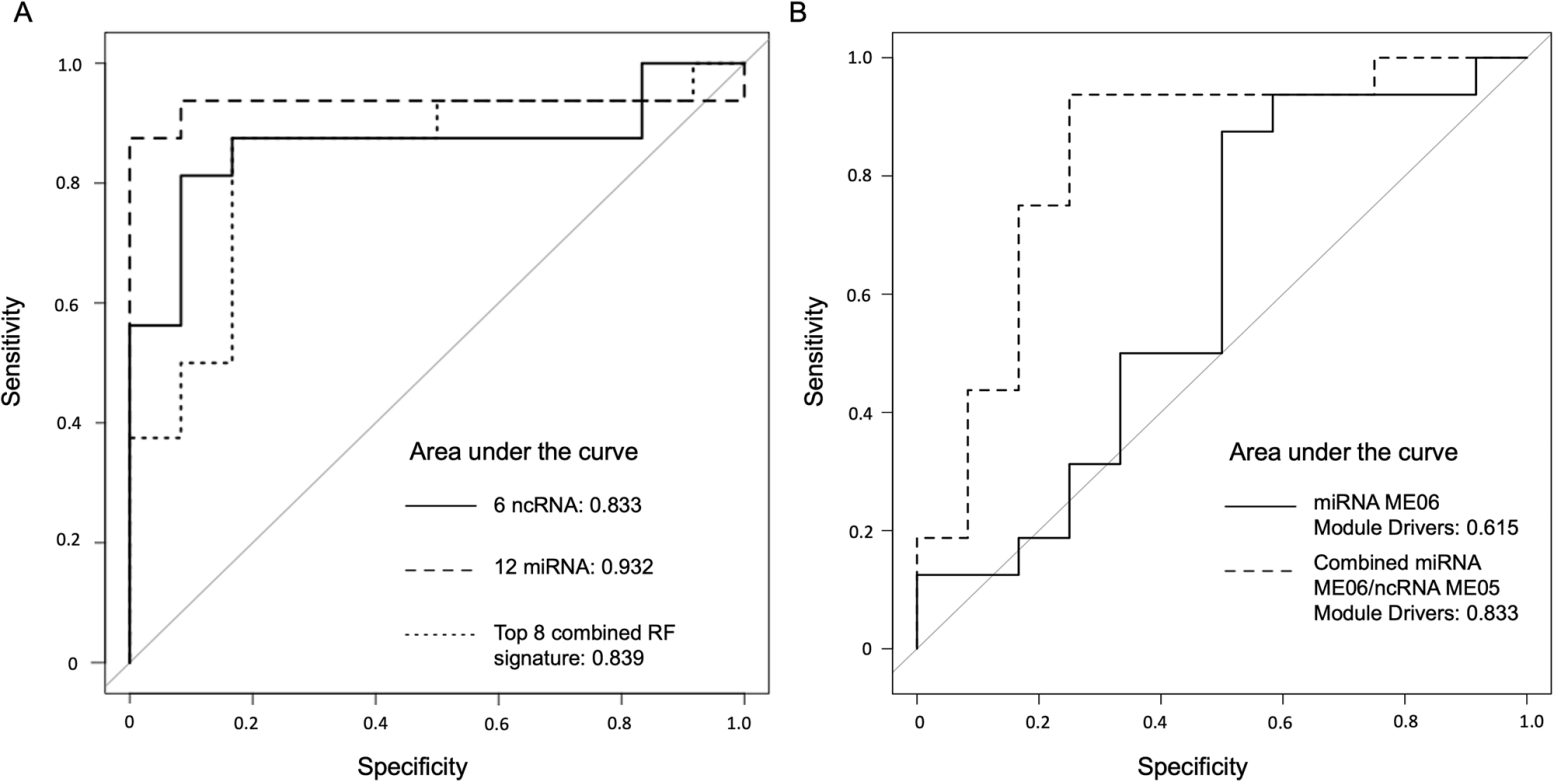
Receiver operating characteristic (ROC) curves for the derived L1EV small RNA signatures in differentiating patients with AIS. (**A**) Performance of ncRNA, miRNA, and a combined small RNA signature derived by random forest classification and assessed with tenfold nested cross validation; and (**B**) performance of two signatures derived from weighted gene co-expression network analysis.

We performed weighted gene correlation network analysis (WGCNA) of miRNA and other ncRNA expression data and identified 19 miRNA transcript modules and 32 ncRNA module eigengenes (MEs) reflecting significant co-expression. MEs represent the first principal component of the module obtained by singular value decomposition and can be considered representative of the miRNA expression profile of a ME. These identified MEs are arbitrarily designated as ME01-ME19 for miRNAs and ME01-ME32 for other ncRNAs (Supplemental Fig. 3A,B). WGCNA can provide pathophysiological insights into high-dimensional transcriptomic data by constructing a gene network based on correlations in sample expression profiles. miRNAs that are highly co-expressed in the network are subsequently grouped into MEs. The resulting modules of miRNAs may better reflect physiological functions, thus MEs represent coherent transcriptomic signatures in L1EVs that covary with patient characteristics and are therefore targets for biomarker generation. We performed correlation analysis between MEs and clinical variables (see Fig. 3 and Supplementary Fig. 3C,D). Among the identified miRNA modules, ME12 exhibited the highest correlation with initial National Institutes of Health Stroke Scale (NIHSS; Spearman ρ = 0.56; *P* value = 0.002) as well as large artery atherosclerosis (LAA) stroke subtype; ME10 demonstrated a trend towards inverse correlation with diagnosis of AIS (Spearman ρ = − 0.35; *P* value = 0.07). Specific correlations were also observed between miRNA expression modules and hyperlipidemia (HLD) (ME01, ME06, and ME10), smoking (ME13 and ME15; see Fig. 2) prior antiplatelet/anticoagulant treatment (ME 14), and type 2 diabetes (T2DM, ME11; see Supplementary Fig. 3C). Correlations with other stroke subtypes are unlikely to be meaningful given the small sample size. Among the identified ncRNA modules ME05 exhibited the strongest correlation with diagnosis (Spearman ρ = − 0.50; *P* value = 0.007) and ME10 the strongest correlation with NIHSS (Spearman ρ = 0.47; *P* value = 0.01), No ncRNA module was associated with sex. Significant correlations were observed between clinical variables and non-overlapping ncRNA MEs including: HLD (ncRNA ME08 and ME19; smoking (ncRNA ME20); prior antiplatelet/anticoagulant (ME26), T2DM (ME25), and interestingly several were uniquely associated with age (ME02, ME05, ME08, ME13, ME16, and ME29).

In WGCNA, high module membership (MM) alone identifies hub genes in a module, whereas those genes showing high MM and high gene significance (GS) for a clinical variable can potentially be considered as drivers. The sign of module membership encodes whether the gene has a positive or a negative relationship with the ME. Significant driver miRNAs of modules correlated with initial NIHSS score and diagnosis are listed in Table 2. The driver genes for the miRNA module associated with NIHSS (ME12) were hsa-miR-28-3p (MM: 0.79, GS *P*-value: 0.003), hsa-miR-126 (MM: 0.93, GS *P*-value: 0.007), hsa-miR-125a-5p (MM: -0.54, GS *P*-value: 0.013), and hsa-miR-301a-3p (MM: 0.84, GS *P*-value: 0.015). The top drivers for miRNA modules with a trend towards correlation with diagnosis (ME10) were hsa-miR-154-3p (MM: − 0.36, GS *P*-value: 0.004), hsa-miR-4448 (MM: 0.78, GS *P*-value: 0.009), and hsa-mir-876-3p (MM: 0.64, GS *P*-value: 0.012. Notably, ncRNA module drivers of ME05, which was significantly associated with diagnosis includes: LINC02868 (MM: 0.65, GS *P*-value: 0.002), LINC01484 (MM: − 0.75, GS *P*-value: 0.003), lnc-SBDS-11 (MM: − 0.58, GS *P*-value: 0.003), and LINC02093 (MM: 0.65; GS *P*-value: 0.018). While top drivers of ME10, which correlated with NIHSS, were lnc-ASTN2-1 (MM: 0.66; GS *P*-value: 0.0004), PANCR (MM: 0.81; GS *P*-value: 0.0005), LINC02579 (MM: 0.80; GS *P*-value: 0.0007), and lnc-DSCC1-1 (MM: 0.72; GS *P*-value: 0.001).

**Table 2:** Driver genes for key miRNA modules from WGCNA.

Module Eigengene	Correlated clinical variable	Driver RNA transcripts	Module membership	Gene significance (GS)	GS *P*_*-*_value
ME12	NIHSS	hsa-miR-28-3p	0.79	0.54	0.003
		hsa-miR-126	0.93	0.50	0.007
		hsa-miR-125a-5p	− 0.54	− 0.46	0.013
		hsa-miR-301a-5p	0.84	0.46	0.015
ME10	Diagnosis	hsa-miR-154-3p	− 0.36	0.53	0.004
		hsa-miR-4448	0.78	− 0.48	0.009
		hsa-miR-876-3p	0.64	− 0.47	0.012

We applied random forest (RF) classification and assessed conditional variable importance (see Supplemental Fig. 4) to select a reduced miRNA and miRNA-ncRNA classifier. To appropriately identify and assess the performance of the classifiers in this small cohort, we utilized nested cross-validation^24^. A 12 miRNA signature derived from RF filtering in nested cross-validation showed impressive accuracy with a sensitivity of 87.5% and specificity of 100% (area under the curve (AUC) = 0.932, accuracy 92.7%; Fig. 3A). A similarly derived six ncRNA signature showed a sensitivity of 93.8% and specificity of 91.7% (AUC = 0.833, accuracy 85.7%). Combining the miRNAs and ncRNAs upregulated by AIS from the two signatures, which may have implications for a point-of-care diagnostic test, we found comparable performance with a smaller 8 small RNA signature (sensitivity of 87.5% and specificity of 83.3%, AUC = 0.839, accuracy 85.7%). This 8 small RNA signature performed similarly in males and females (Supplementary Fig. 6A). We also took advantage of the WGCNA modules as an unbiased means of gene signature identification and compared the performance of module drivers of diagnosis to RF classification (Fig. 3B). Performance of the miRNA module drivers of ME06 in classifying AIS by tenfold cross validation was poor (AUC 0.615, Accuracy 67.9%), but the combination miRNA ME06 and ncRNA ME05 module drivers (7 small RNAs) performed comparably to the 8 small RNA signature specified by RF models (AUC 0.833, Accuracy 75.0%; see Fig. 3B). The combined miRNA/ncRNA module driver signature also performed similarly in males and females (Supplementary Fig. 6B). Expression levels of the key diagnostic signature miRNAs and ncRNAs across cohorts using next-generation sequencing are shown in the Supplemental Fig. 5. Validation of predictive miRNAs by quantitative real-time (qRT)-PCR was performed for the 5 miRNAs in the 8 small RNA signature, and significant upregulation was observed in 4 of the 5 species of miRNAs including hsa-miR-27b-3p, hsa-miR-154-3p, hsa-miR-221-3p, and hsa-miR-181a-5p (see Supplementary Fig. 6C).

## Discussion

Our results indicate that L1EV miRNA and other ncRNA fragment expression patterns can distinguish AIS patients from patients without stroke. Moreover, miRNA and other ncRNA modules identified in WGCNA are also significantly associated with NIHSS scores among other clinical variables, which may help guide future studies regarding the pathophysiological underpinnings of these associations. Using RF modeling, we established an 8 small RNA signature that classified AIS with excellent accuracy. Confirmation in larger patient cohorts will be important in validating and refining these findings.

Our approach offers several strengths compared to previous efforts to identify blood-based biomarkers of AIS. First, we analyzed L1EVs by isolating EVs from plasma binding to L1CAM. Second, we performed small RNA sequencing and comprehensively aligned reads to the latest small RNA databases. Third, we pursued unbiased analyses and generated minimal miRNA/ncRNA signatures using the RF nested cross-validation with the aim of providing a reproducible biomarker signature. This small RNA sequencing readout of the L1EV response during AIS may also help inform new hypotheses regarding the pathophysiology of stroke and recovery.

Many of the differentially expressed miRNAs or those identified to be module drivers of diagnosis have previously been implicated in AIS including miR-27b, miR-125a-5p, miR-125b-5p, miR-126, and miR-4448^11,25–27^. These miRNAs are highly expressed in brain tissue and/or arteries providing a rationale for further studying their pathophysiological functions in AIS^22^. For example, miR-27b has been strongly linked to several aspects of endothelial function including angiogenesis, oxidative stress, and shear stress^28,29^ and expanding our knowledge of its role in animal models of strokes could provide novel therapeutic insights. Due to the high throughput nature of our approach, there are dozens of differentially expressed miRNAs and other small ncRNAs that have not been previously reported as potential AIS biomarkers in human studies. Many are predicted to target genes critical to brain injury such as VEGF, BDNF, and HIF1a and have been found to have roles in vascular biology, inflammation, or cell stress pathways in preclinical models or translational studies such as miR-185^30^, miR-107^31^, miR-181a^32^, miR-221^33^, and miR-376a^34^. Notably, some differentially expressed ncRNAs identified in AIS converge upon differentially expressed miRNAs targets, hsa-miR-19b-1 and hsa-miR-320a, which have been implicated in stroke risk, atrial fibrillation, and coronary artery disease^35–37^. These miRNAs as well as hsa-miR-185 and hsa-miR-107 are known to target genes involved in hypoxia-related responses, neuro-repair, and angiogenesis including HIF1a, FKBP-5, BDNF, and VEGFa suggesting plausible mechanisms linking L1EV to AIS. Gene ontology analyses of target genes of differentially expressed miRNAs revealed additional intriguing insights. Among the pathways targeted by miRNAs upregulated in AIS are those associated with the mitochondrial outer membrane, apoptotic signaling, and cytokine-mediated signaling. Speculatively, involvement of these pathways suggests that AIS-induced changes in circulating L1EV cargoes may mirror neuronal responses occurring in response to ischemia. Further understanding the relationship between the presence of these miRNAs in L1EVs and their functional roles might provide clues to new therapeutic targets.

Prior biomarker studies have also found other small ncRNAs such as lncRNA, tRNA, snRNA, and snoRNA in exosomes from serum, plasma, or other biofluids^38,39^. Key L1EV ncRNAs identified here were able to classify AIS with similar performance as miRNAs, and when combined with miRNAs additional diagnostic accuracy was achieved with fewer predictors. The physiological role of these other small ncRNA cargoes in EVs is unclear. Interestingly, many miRNA and ncRNA modules were associated with clinical variables such as aging, sex, T2DM, HLD, smoking status, and prior use of antiplatelet or anticoagulant medications. Further investigating RNAs driving these associations could provide pathophysiological clues into how these risk factors affect neuronal health, stroke risk, and neurorecovery. The overlapping correlations of ME10 with diagnosis of AIS and HLD may point to a particularly salient role for ME10 miRNAs in driving risk for both diagnoses. Interestingly, hsa-miR-92a-3p, a ME10 module member for both stroke and HLD (see Supplementary Table 5), is elevated in patients with essential hypertension and carotid stenosis and has been implicated in regulating arterial stiffness^40–42^. In addition, the specific association of ME14 with prior antiplatelet or anticoagulant therapy suggests regulation of critical members of ME14 by these drugs. Indeed, the ME14 miRNA with the highest gene significance score for prior antiplatelet/anticoagulation treatment, hsa-miR-145-3p, has been implicated in aspirin action and thrombus formation (see Supplementary Table 5)^43,44^. Further studies are required to fully elucidate the mechanisms of how specific stroke risk factors modulate L1EV miRNA cargoes, but these data suggest some key candidate miRNAs. Overall, our data align with prior studies of EVs in other diseases indicating the potential of these classes of small RNA, to serve as biomarkers of disease^45,46^. Differentially expressed small ncRNA fragments identified here (RNU-888P and LINC02116) as well as ncRNA module drivers correlated to NIHSS and diagnosis (PANCR and LINC01484), have also been connected to stroke or stroke risk factors in prior studies^47–50^.

It is important to note the limitations of our study. The sample size is small and further study in larger cohorts is necessary. Our population of AIS patients was enriched in minor stroke, and further study in populations with the breadth of ischemic injuries (i.e. transient ischemic attack and larger territory AIS) will be of great interest. The rationale for this choice in our pilot study is to avoid marked variability in pre-existing disabilities and comorbidities between a control population. Another consequence of the small sample size is that stroke subtype-specific classification analyses were not possible. In addition, we were not able to include quantitative imaging data in the development of a L1EV small RNA signatures, which could offer more detailed insights into patients’ conditions and enable more accurate predictions. Although we were able to validate miRNA targets selected by the random forest model in qRT-PCR, an important limitation to note is a miRNA reference gene for specific EVs has not been extensively studied. In our next-generation sequencing, hsa-miR-138-5p was among the top 1% of genes with lowest variance across all samples. One prior study also used hsa-miR-138-5p as a reference gene for biomarker-related EV miRNA qRT-PCR^51^. However, its choice remains a potential source of bias and further studies are required to determine a reliable L1EV reference miRNA prior to establishing a diagnostic test. As we increase the number of enrolled patients with detailed acute stroke imaging and sequenced EV samples, such analyses and establishment of a reliable reference gene will be feasible. A future goal of our ongoing work is to predict stroke etiology and long-term outcome in larger multi-center studies to provide a basis for precision secondary stroke prevention. Pathophysiologically, many unanswered questions remain regarding the mechanisms underlying regulation of EV cargoes derived from ischemic or injured central nervous system cells and whether centrally derived circulating EVs play a role shaping the immune response by signaling injury. In addition, L1CAM is likely not specific to neuronally-derived EVs, thus further defining high-fidelity markers of neuronally-derived EVs (as well as other cell-type specific EVs) will increase the reliability and reproducibility of future EV biomarker studies^6,20^. Interestingly, ATPase Na+/K+ transporting subunit alpha 3 (ATP1A3) has recently emerged as a more promising neuron-specific EV marker, as ATP1A3-EV cargoes are associated with greater specificity for neurons than L1EVs^52^. Further investigating the utility of ATP1A3-EVs, which also express L1CAM, as a biomarker of neurological disease such as AIS may provide even greater diagnostic and prognostic capability.

Our results indicate that L1EVs isolated from plasma contain miRNA as well as other ncRNA cargoes. The most abundant miRNAs present in plasma L1EVs are typically expressed in the brain. Stroke alters the expression pattern of miRNA and other ncRNA in L1EV from plasma in our cohort of stroke patients. The utility of these small RNA signatures in diagnosing stroke will require validation in larger cohorts. In addition, further understanding the functional role of miRNA and ncRNA L1EV cargoes in response to brain injury may provide important insights into neurorecovery and repair processes.

## Methods

### Cohort selection and biospecimen collection

Patients with AIS or cardiovascular disease were enrolled at Memorial Hermann Hospital or UT Physicians cardiology clinic, UT-Health, Houston, Texas, USA. The inclusion criteria for patients with AIS were age > 18 years, blood specimens drawn < 24 h after admission, AIS confirmed by imaging, and the absence of known malignancy or autoimmune disease. The inclusion criteria for control patients were age > 18 years, no diagnosis of AIS, absence of known malignancy or autoimmune disease, and the presence of at least 3 stroke risk factors (age > 65, T2DM, HTN, atrial fibrillation, or hyperlipidemia). The clinical information about the patients and controls was obtained through review of electronic medical records and patients' self-report. All the methods and protocols presented in this study were approved by the Institutional Review Board of the University of Texas-Houston and University of California, San Francisco (HSC-MS-17-0452 and #19-27658). All participants provided written informed consent before enrollment. All research was performed in accordance with local regulations as well as with the Declaration of Helsinki.

Peripheral blood was collected in sterile vacutainers. Blood plasma was isolated by centrifuging samples at 1200*g* for 10 min at 4 °C, followed by plasma supernatant isolation and further centrifugation at 10,000*g* for 10 min at 4 °C to generate plasma. Samples were then aliquoted and stored at − 80 °C until analysis.

### EV and miRNA isolation

L1EV isolation was performed as previously described^53^. Briefly, 250 µl of plasma was incubated with 100 µl of thromboplastin-D (Fisher Scientific, Hanover Park, IL) followed by the addition of 150 µl of calcium- and magnesium-free Dulbecco’s balanced salt solution with protease and phosphatase inhibitor cocktail (Roche Applied Science, Indianapolis, IN). ExoQuick (System Biosciences, Mountainview, CA) was added to precipitate total EVs, that were re-suspended for immunochemical enrichment of EVs from neural sources. L1EVs were obtained by sequential immunoprecipitation with biotinylated monoclonal antibody to L1 cell adhesion molecule (L1CAM; eBioscience, San Diego, CA). Nanosight NS300 instrument (Malvern Instruments, Malvern, UK) in combination with an EV membrane label, ExoGlow (System Biosciences, Palo Alto, CA) confirmed that the particle sizes of total EV extracts were within range for exosomes (30–220 nm). To determine EV purity per total protein of sample, plasma protein concentration was measured by spectrophotometer at 562 nm after Pierce Bicinchoninic Acid Protein Assay (ThermoFisher, Waltham, MA). Small RNA enriched total RNA was isolated from each sample using Acid-Phenol:Chloroform extraction (*mir*Vana PARIS Kit, Invitrogen, Waltham, MA).

### RNA-sequencing and qRT-PCR

75 ng of extracted RNA was used for the library synthesis. Small RNA was enriched, purified, and cDNA libraries synthesized by a commercial vendor (BGI America, San Jose, CA). Adaptor and unique molecular identifier sequences were added. The libraries were quantified and quality control measured using a Bioanalyzer (Agilent). Barcoded libraries were pooled together and sequenced. After eliminating low-quality reads and adaptors, each sample generated over 20 million clean reads (Supplementary Table 1).

To quantify the expression of miRNAs MystiCq miRNA cDNA Synthesis kit (Sigma-Aldrich, USA) was used to prepare cDNA for miRNAs in each RNA sample. Quantitative PCR was performed with miScript SYBR Green PCR Kit (QIAGEN, Germantown, MD) using a universal MystiCq reverse primer and a forward primer specific for each different miRNA assayed (hsa-miR-27b: UUCACAGUGGCUAAGUUCUGC; hsa-miR-154-3p: AAUCAUACACGGUUGACCUAUU; hsa-miR-376a-3p: AUCAUAGAGGAAAAUCCACGU; hsa-miR-221-3p: AGCUACAUUGUCUGCUGGGUUUC; hsa-miR-181a-5p: AACAUUCAACGCUGUCGGUGAGU; and hsa-miR-138-5p: AGCUGGUGUUGUGAAUCAGGCCG). hsa-miR-138-5p was chosen as a control based on having a very low variance across all samples in next-generation sequencing and its prior use as a control in EV miRNA qPCR^51^. Real-time qPCR was run with the LightCycler 96 instrument (Roche Life Science, Indianapolis, IN) and cycling conditions were as follows; 5 min at 95 °C followed by 45 cycles of 15 s at 95 °C and 1 min at 60 °C. Expression levels were calculated using ΔΔCT method with hsa-miR-138-5p for normalization. Statistical significance was calculated in R using two-tailed Student T-test.

### Bioinformatic and statistical analysis

Data analysis was performed in R^54^ using the statistical packages that are specifically mentioned below as well as the packages tidyverse^55^, dplyr^56^, ggplot2^57^, and EnhancedVolcano^58^. The raw reads of the fastq files were tested for quality control using the FastQC software^59^ and were then aligned to the human reference genome (hg38 from the University of California, Santa Cruz) using the MANATEE small RNA processing pipeline^60^ to summarize the miRNA and other ncRNA counts. The programs DESeq2 and limma on the iDEP platform were used for normalization and differential expression analysis of miRNA and other ncRNA counts^61–63^.

The normalized expression matrix generated for the differential gene expression analysis with DESeq2 was used for additional downstream analyses. Tissue enrichment and gene ontology pathway analysis was carried out with online tools: miRNA Enrichment Analysis and Annotation (miEAA) tool 2.1 and miRTargetLink 2.0^23,64^. Genes annotated to be strongly linked by experimental evidence to the differentially expressed miRNAs were used as the input for gene ontology (GO) analysis with Enrchr-KG using GO: Biological Pathways, Cellular Component, and Molecular Function databases^65^. Interactions between differentially expressed miRNAs and lncRNAs were curated with miRNA-lncRNA module of DIANA tools LncBase v3^66^. The Sankey diagram was generated with the R package networkD3^67^.

The WGCNA package was used to obtain MEs (module eigengenes)^68^. MEs represent the first component of the module obtained by singular value decomposition and were correlated with clinical variables using Pearson correlation analysis. The absolute correlation between the ME with each RNA expression profile determines the MM (module membership). Thus, a MM value close to 1 for a given gene indicates a good representative of the module (gene hub). RNAs significantly associated with a particular clinical variable (termed GS: gene significance) and with high MM are considered potential driver genes. For each clinical variable and module, we ranked the genes according to the highest MM and GS values. Using one-way ANOVA with Tukey’s multiple comparison correction, we identified gene modules that were highly specific for clinical variables in the data set.

We generated a predictive model of AIS etiology using gene drivers from the WGCNA modules and the normalized expression matrix. Diagnosis of AIS was used as the target outcome variable in binomial a logistic regression model. In addition, to provide a robust ncRNA signature detection process in the setting of the low number of samples and imbalance between groups of this study, tenfold nested cross-validation was performed with RF filtering for variable selection^69^. Nested cross-validation partitions the dataset into inner and outer folds to recursively test and tune predictors. The inner fold data is used to tune parameters and fit the model, which is repeatedly tested on the left-out data from the outer fold. The test predictions from the outer folds are compared against the true results for the outer test folds and the results are concatenated, to give measures of accuracy and area under receiver operating characteristic (AUROC). The resulting classification models were tuned, specified, and assessed using glmnet, randomforestsrc, caret, and nestedcv packages in R^24,69–71^.

### Supplementary Information

Supplementary Figures.

Supplementary Table S1.

Supplementary Table S2.

Supplementary Table S3.

Supplementary Table S4.

Supplementary Table S5.

## Data Availability

Anonymized data and materials are publicly available at NCBI Gene Expression Omnibus (GSE 269195).
