# Supplementary material for: Small RNA signatures of acute ischemic stroke in L1CAM positive extracellular vesicles
Source: Sci Rep. 2024 Jun 12;14:13560. doi: 10.1038/s41598-024-63633-4 (PMC11169361; doi:10.1038/s41598-024-63633-4)
Supplement: Supplementary file 1 — Supplementary Figures. [file 41598_2024_63633_MOESM1_ESM.pdf]

SUPPLEMENTAL FIGURE 1

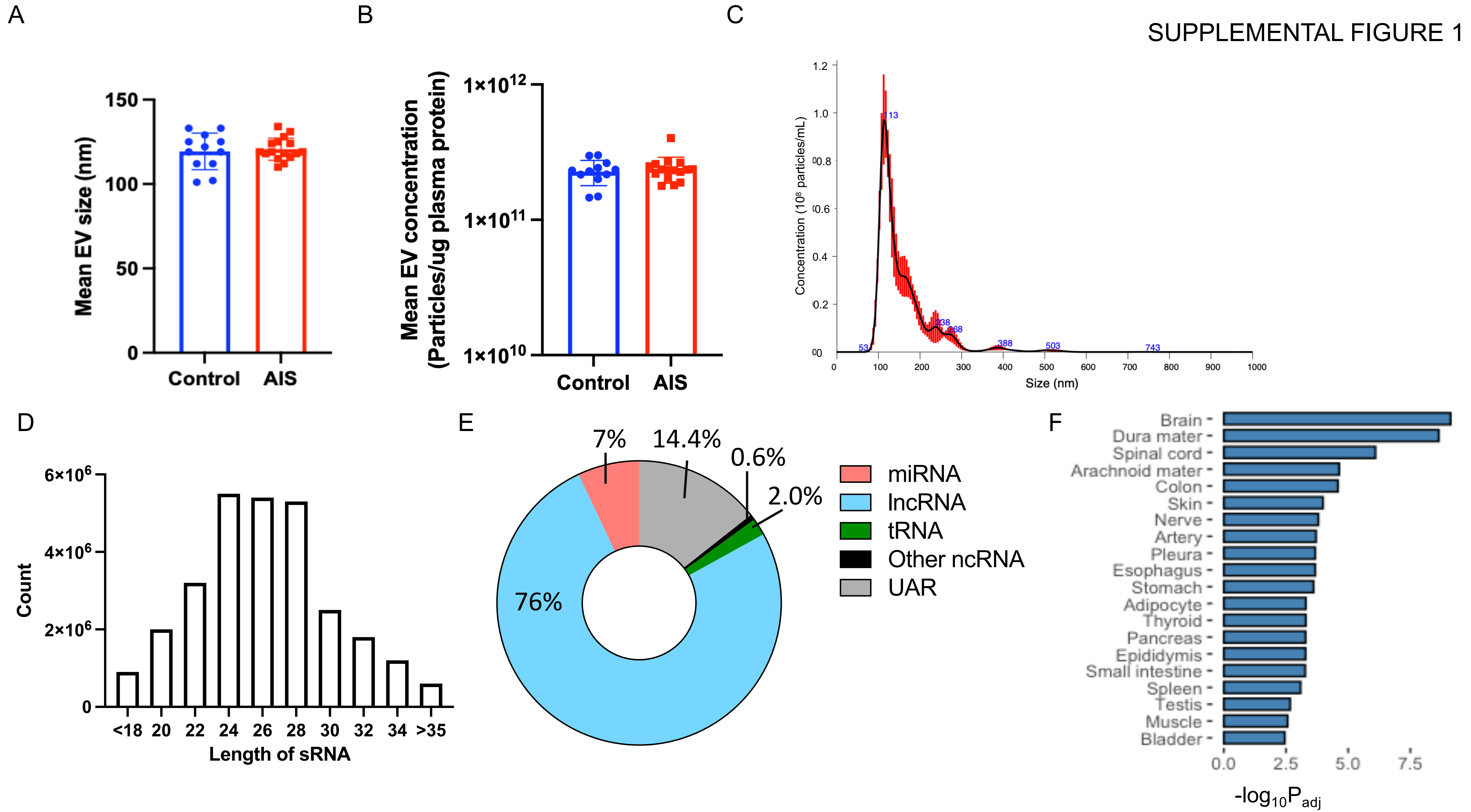

Distribution of small RNA samples: (A): Mean L1EV size determined by nanoparticle tracking analysis. **(B):** Mean L1EV concentration per ug plasma protein. **(C):** Representative nanoparticle size distribution obtained by nanoparticle tracking analysis. **(D):** Histogram demonstrates the length composition of the small RNA samples. Most small RNA is found to be between 18-30 nucleotides (nt). The microRNAs (miRNAs) are located around the main peak at 22-24 nt; Other non-coding (nc)RNAs are longer including piwi-interacting RNAs, Y4-RNA, transfer RNA, small nucleolar RNA, mRNA, circular RNA, and fragments of long intergenic non-coding (lnc)RNA. **(E)** Small RNA species subtype as determined by alignment of sequencing reads to small RNA databases corroborates the samples are enriched in lincRNA, miRNA, and tRNA; but also contain a small percentage of other small RNA species. UAR: unannotated reads. **(F):** miRNA tissue enrichment analysis: the top 65 expressed miRNAs in the L1EVs of all patients were used as input in the miRNA Enrichment Analysis and Annotation (miEAA) tool (Aparicio-Puerta et al., 2023).

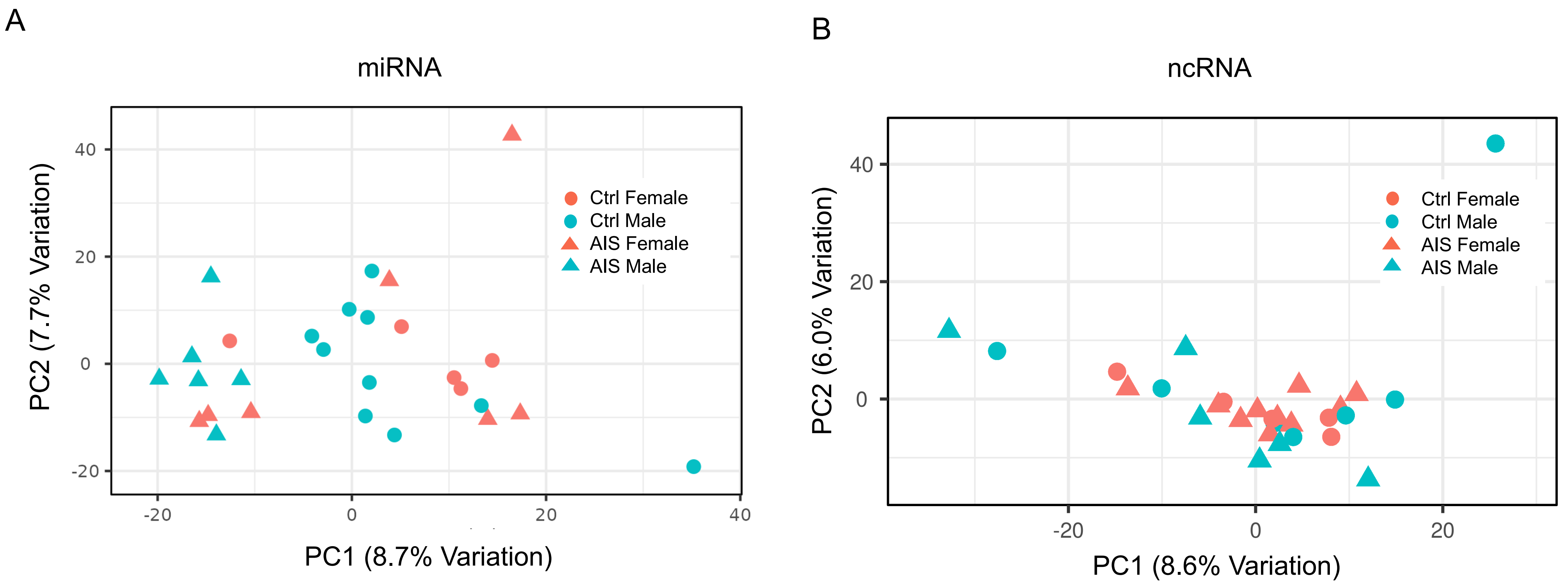

Principal Component Analysis of neuron-derived exosomes from plasma of acute ischemic stroke (AIS) patients vs. stroke-mimic patients reveals segregation in variance by diagnosis and sex in (A) miRNA and (B) lncRNA normalized expression data.

SUPPLEMENTAL FIGURE 2

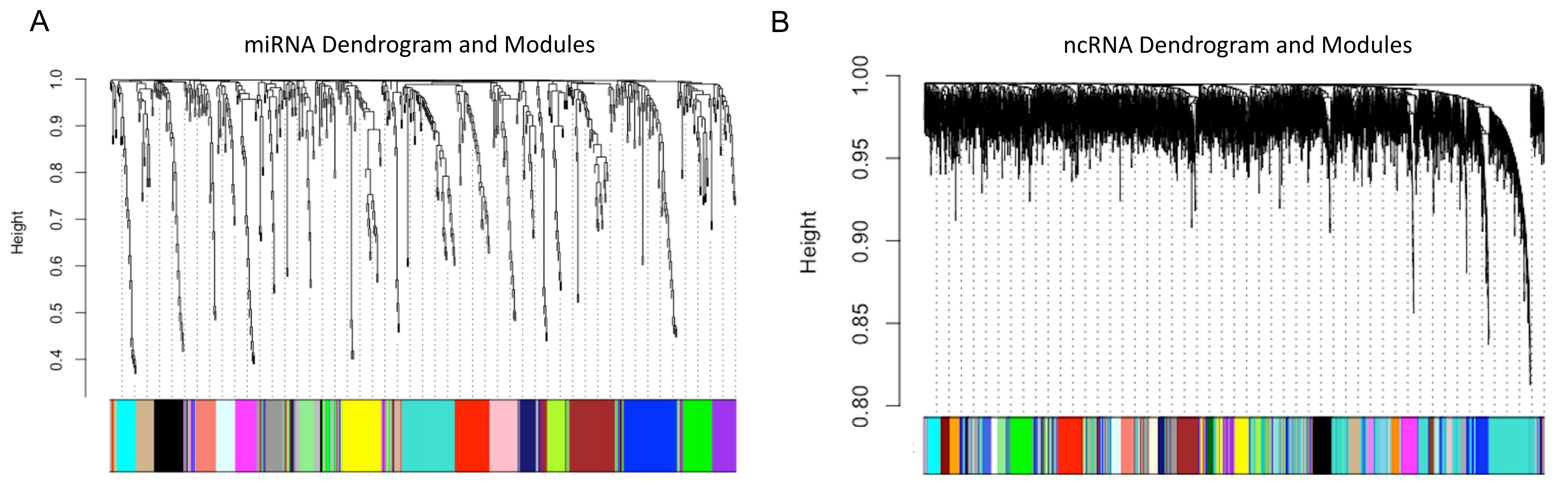

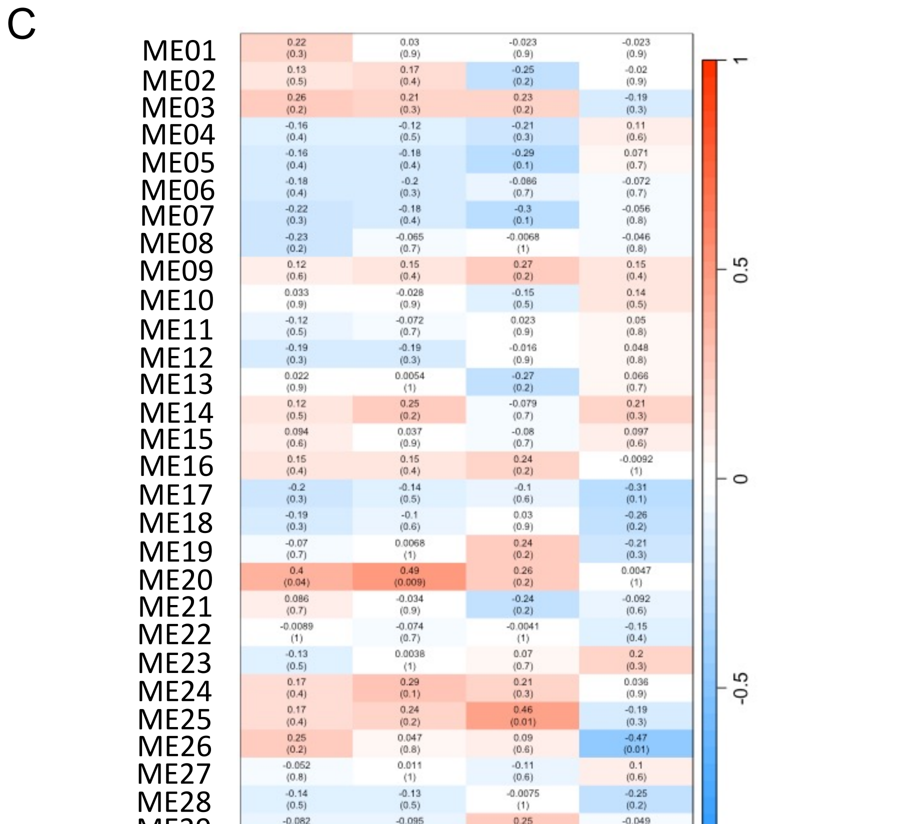

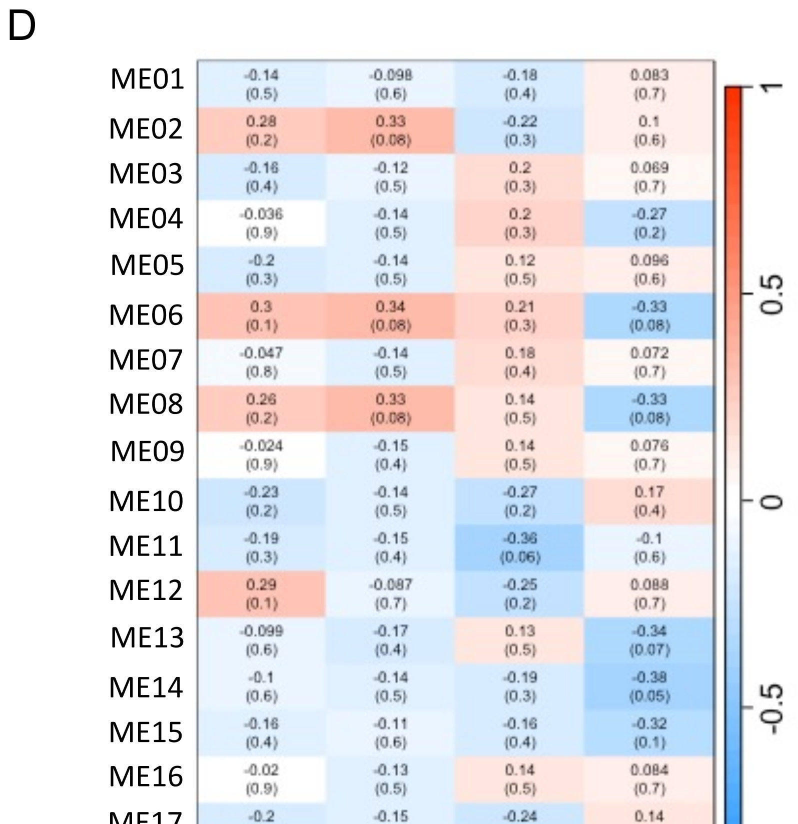

Cluster Dendrogram of (A) miRNA and (B) other ncRNA fragments demonstrating weighted gene correlation network analysis (WGCNA). RNAs demonstrating correlated expression are clustered in modules represented by colors below the dendrogram. miRNAs are grouped into 19 modules; lncRNA fragments are grouped into 32 modules. (C) miRNA and (D) other ncRNA correlation heat map of gene modules correlation with clinical variables of interest.

SUPPLEMENTAL FIGURE 3

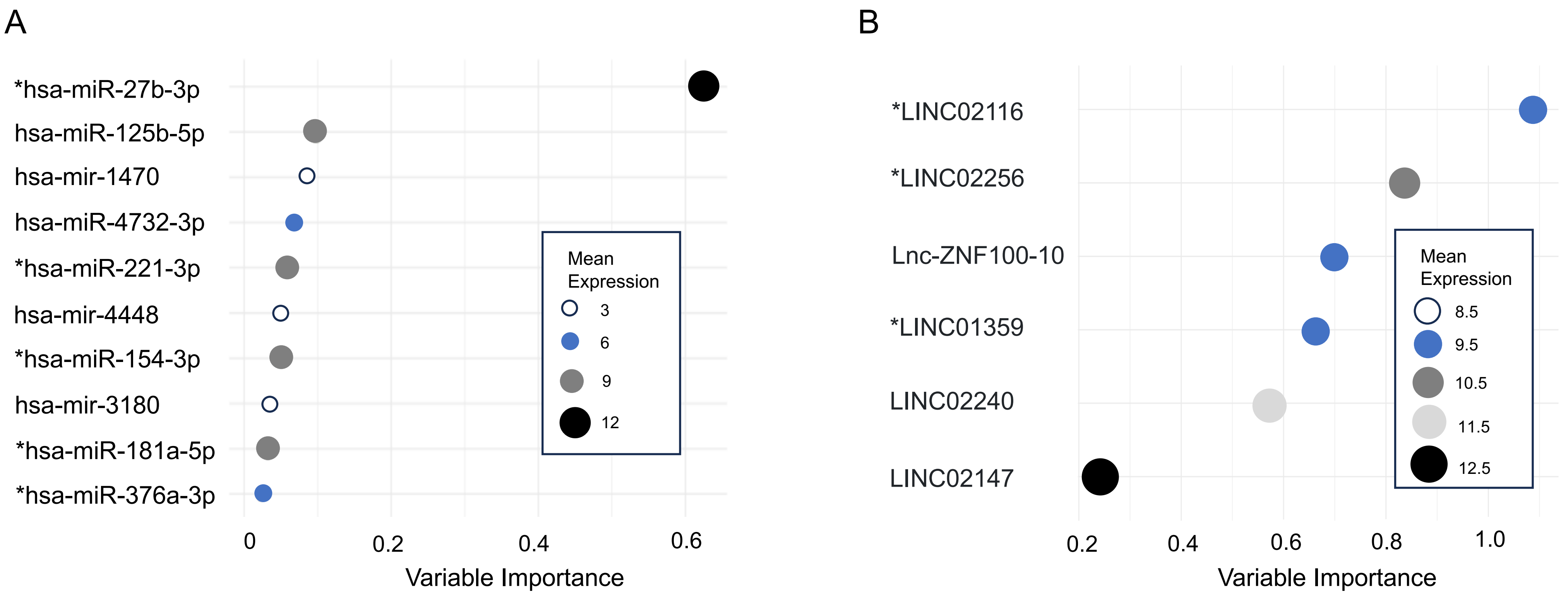

Random forest variable importance analysis. (A) miRNA and (B) other ncRNA signature predictors of AIS were derived by variable importance analysis. Shown are the most salient random forest predictors in classifying AIS as well as the mean normalized expression level. * indicates the small RNAs combined on the basis of variable importance and demonstrating upregulation in AIS for the combined 5 small RNA signature.

SUPPLEMENTAL FIGURE 4

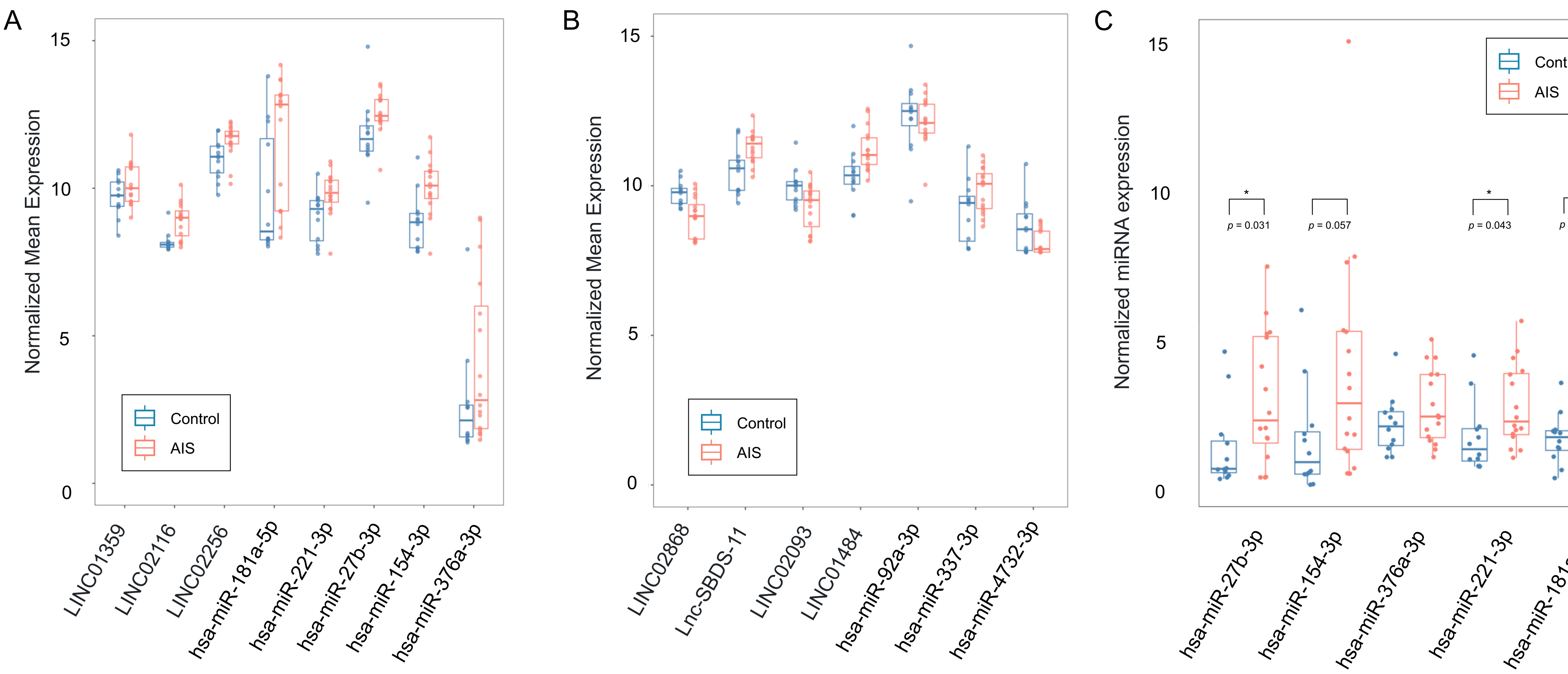

Normalized mean expression of selected small RNAs and RT-qPCR validation. Boxplot demonstrating median (solid line) and interquartile range (box) of small RNA normalized expression of (A) 8 miRNA/ncRNA random forest-derived RNA signature up-regulated in AIS**;** (B) module drivers of diagnosis from weighted gene correlation [illegible] analysis; and (C) RT-qPCR validation of 5 random forest derived miRNA species normalized to hsa-miR-138-5p.

SUPPLEMENTAL FIGURE 5

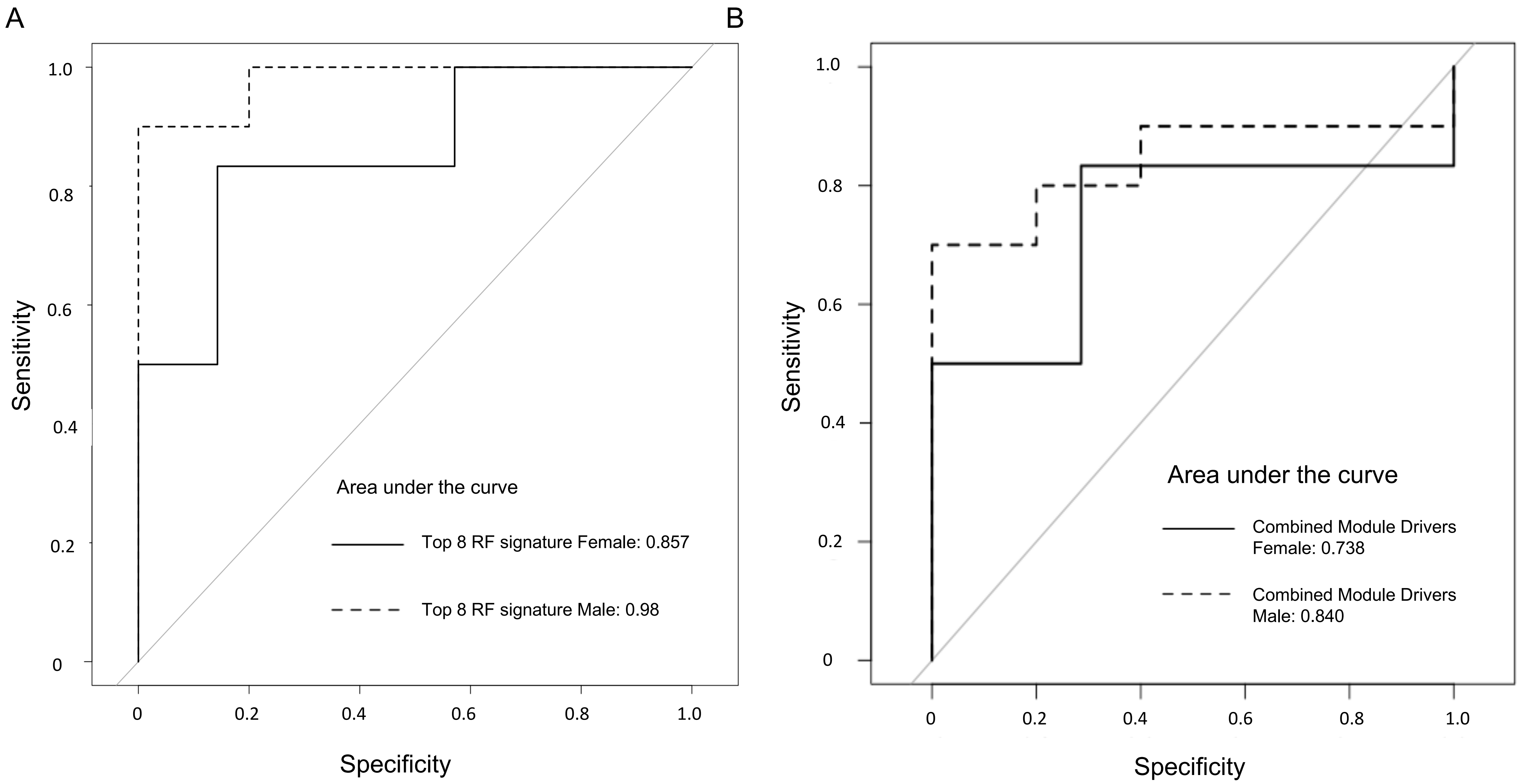

ROC curves for the Random Forest (RF) and weighted gene correlation network analysis (WGCNA) small RNA signatures in Males and Females. (A) Performance of a eight small RNA signature derived and assessed by nested RF classification and 10-fold cross validation; and **(B)** performance of miRNA ME06 drivers alone and combined with ncRNAs ME05 drivers derived from WGCNA in males and females.

SUPPLEMENTAL FIGURE 6
