# Supplementary material for: Small RNA signatures of acute ischemic stroke in L1CAM positive extracellular vesicles
Source: Sci Rep. 2024 Jun 12;14:13560. doi: 10.1038/s41598-024-63633-4 (PMC11169361; doi:10.1038/s41598-024-63633-4)
Supplement: Supplementary file 2 — Supplementary Table S1. [file 41598_2024_63633_MOESM2_ESM.docx]

Table S1. **L1EV and sequencing output and quality metrics**

|  | Control  (*n* = 12) | Acute ischemic stroke  (*n* = 16) | Overall  (*n* = 28) | |
| --- | --- | --- | --- | --- |
| Particles/µg protein |  |  |  | |
| Mean ± SD | 2.27 x 10^11^ ± 4.83 x 10^10^ | 2.38 x 10^11^ ± 5.20 x 10^10^ | 2.37 x 10^11^ ± 4.98 x 10^10^ | |
| Number of reads (in millions) |  |  |  | |
| Mean ± SD | 33.4 ± 6.52 | 33.6 ± 5.00 | 33.5 ± 5.57 |  |
| Median [Min, Max] | 30.7 (27.8, 48.0) | 32.2 (27.7, 46.2) | 31.6 (27.7, 48.0) |  |
| **Clean Reads (in millions)** |  |  |  |  |
| Mean ± SD | 24.6 ± 1.08 | 24.4 ± 1.14 | 24.5 ± 1.10 |  |
| Median [Min, Max] | 24.0 (23.9, 26.9) | 24.0 (22.3, 27.5) | 24.0 (22.3, 27.5) |  |
| **% ≥Q20 bases** |  |  |  |  |
| Mean ± SD | 97.5 ± 0.452 | 97.8 ± 0.226 | 98.6 ± 0.12 |  |
| Median [Min, Max] | 98.6 (98.3, 98.7) | 98.6 (98.3, 98.8) | 98.6 (98.3, 98.8) |  |
