# Supplementary material for: Small RNA signatures of acute ischemic stroke in L1CAM positive extracellular vesicles
Source: Sci Rep. 2024 Jun 12;14:13560. doi: 10.1038/s41598-024-63633-4 (PMC11169361; doi:10.1038/s41598-024-63633-4)
Supplement: Supplementary file 3 — Supplementary Table S2. [file 41598_2024_63633_MOESM3_ESM.docx]

Table S2. **Top expressed miRNA in isolated L1EVs of all samples.**

| miRNA | Log CPM | Brain Expression* |
| --- | --- | --- |
| \| \| hsa-miR-204-5p \| \| --- \| \| hsa-miR-92b-3p \| \| hsa-miR-127-3p \| \| hsa-miR-451a \| \| hsa-miR-139-5p \| \| hsa-miR-125a-5p \| \| hsa-miR-149-5p \| \| hsa-miR-124-3p \| \| hsa-miR-99a-5p \| \| hsa-let-7b-5p \| \| hsa-miR-222-3p \| \| hsa-miR-92a-3p \| \| hsa-miR-27b-3p \| \| hsa-miR-9-5p \| \| hsa-let-7i-5p \| \| hsa-miR-24-3p \| \| hsa-miR-150-5p \| \| hsa-miR-181a-5p \| \| hsa-miR-22-3p \| \| hsa-miR-181b-5p \| \| hsa-miR-128-3p \| \| hsa-miR-132-5p \| \| hsa-let-7c-5p \| \| hsa-miR-219a-2-3p \|   hsa-miR-328-3p \| \| --- \| --- \| --- \| --- \| --- \| --- \| --- \| --- \| --- \| --- \| --- \| --- \| --- \| --- \| --- \| --- \| --- \| --- \| --- \| --- \| --- \| --- \| --- \| --- \| --- \| | \| 17.1 \| \| --- \| \| 16.1 \| \| 16.1 \| \| 15.9 \| \| 15.1 \| \| 14.9 \| \| 14.8 \| \| 14.8 \| \| 14.4 \| \| 14.2 \| \| 14.1 \| \| 13.9 \| \| 13.9 \| \| 13.9 \| \| 13.7 \| \| 13.7 \| \| 13.6 \| \| 13.6 \| \| 13.5 \| \| 13.4 \| \| 13.4 \| \| 13.4 \| \| 13.4 \| \| 13.3  13.2 \| | \| Enriched \| \| --- \| \| Enriched \| \| Enriched \| \| Enriched \| \| Enriched \| \| Enriched \| \| Enriched \| \| Uniquely expressed in brain \| \| Enriched \| \| Enriched \| \| Enriched \| \| Enriched \| \| Enriched \| \| Uniquely expressed in brain \| \| Enriched \| \| Enriched \| \| Present in Brain and other tissues \| \| Enriched \| \| Enriched \| \| Enriched \| \| Enriched \| \| Enriched \| \| Enriched \| \| Uniquely expressed in brain \| \| Enriched \| |

*Brain miRNA expression based on Tissue Atlas 2 (42). Classified as: “Uniquely expressed in brain” if the miRNA is only expressed in brain or spinal cord; “Enriched” if the brain is in the top 3 tissues of that miRNA’s expression; or “Present in Brain and other tissues” if the miRNA is ubiquitously expressed in tissues including the brain.
