# Supplementary material for: Small RNA signatures of acute ischemic stroke in L1CAM positive extracellular vesicles
Source: Sci Rep. 2024 Jun 12;14:13560. doi: 10.1038/s41598-024-63633-4 (PMC11169361; doi:10.1038/s41598-024-63633-4)
Supplement: Supplementary file 6 — Supplementary Table S5. [file 41598_2024_63633_MOESM6_ESM.docx]

**Supplementary Table 5. Driver genes for key miRNA modules from WGCNA.**

| **Module Eigengene** | **Correlated Clinical Variable** | **Driver RNA transcripts** | **Module Membership** | **GS** | **GS *P_-_*value** |
| --- | --- | --- | --- | --- | --- |
| **ME14** | **Prior antiplatelet or anticoagulation medication** |  |  |  |  |
|  |  | hsa-miR-145-3p | 0.538 | -0.49 | 0.008 |
|  |  | hsa-mir-219a-1 | 0.587 | -0.44 | 0.017 |
|  |  | hsa-miR-574-3p | 0.578 | -0.42 | 0.027 |
|  |  | hsa-miR-429 | 0.96 | -0.41 | 0.029 |
| **ME10** | **Hyperlipidemia** |  |  |  |  |
|  |  | \| hsa-mir-7706 \| \| --- \| | 0.73 | -0.41 | 0.030 |
|  |  | \| hsa-miR-92a-3p \| \| --- \| | 0.76 | -0.40 | 0.036 |
|  |  | \| hsa-miR-6889-3p \| \| --- \| | 0.60 | -0.50 | 0.006 |
